# Evidence for Retrogene Origins of the Prion Gene Family

**DOI:** 10.1371/journal.pone.0026800

**Published:** 2011-10-27

**Authors:** Sepehr Ehsani, Renzhu Tao, Cosmin L. Pocanschi, Hezhen Ren, Paul M. Harrison, Gerold Schmitt-Ulms

**Affiliations:** 1 Tanz Centre for Research in Neurodegenerative Diseases, University of Toronto, Toronto, Ontario, Canada; 2 Department of Laboratory Medicine and Pathobiology, University of Toronto, Toronto, Ontario, Canada; 3 Department of Biology, McGill University, Montreal, Quebec, Canada; INSERM, UMR-S747, France

## Abstract

The evolutionary origin of prion genes, only known to exist in the vertebrate lineage, had remained elusive until recently. Following a lead from interactome investigations of the murine prion protein, our previous bioinformatic analyses revealed the evolutionary descent of prion genes from an ancestral ZIP metal ion transporter. However, the molecular mechanism of evolution remained unexplored. Here we present a computational investigation of this question based on sequence, intron-exon, synteny and pseudogene analyses. Our data suggest that during the emergence of metazoa, a cysteine-flanked core domain was modularly inserted, or arose *de novo*, in a preexisting ZIP ancestor gene to generate a prion-like ectodomain in a subbranch of ZIP genes. Approximately a half-billion years later, a genomic insertion of a spliced transcript coding for such a prion-like ZIP ectodomain may have created the prion founder gene. We document that similar genomic insertions involving ZIP transcripts, and probably relying on retropositional elements, have indeed occurred more than once throughout evolution.

## Introduction

Prion diseases are fatal neurodegenerative diseases which can affect a relatively broad range of host organisms including humans, sheep, cattle and deer. The normal cellular prion protein, denoted PrP^C^, and coded for by the prion gene (*prnp*), is found in most cell types within the body. In disease, this protein undergoes a structural transition to its disease-causing scrapie form (PrP^Sc^) with profoundly altered physicochemical properties [1]. The accumulation of PrP^Sc^ is toxic to cells and may eventually lead to widespread cell death that is characteristically accompanied by a spongiform degeneration of the brains of afflicted individuals. Despite a wealth of data on the evolutionary conservation, cellular localization, structure, molecular environment and metal-binding properties of PrP^C^, its precise cellular functions are still debated [2].

Extensive genomic investigations have provided evidence for additional PrP-related sequences in the vertebrate lineage [3], [4], [5]. In mammals, two paralogs of the prion gene, the genes encoding for the proteins Doppel/Dpl (*prnd*) and Shadoo/Sho (*sprn*) have been described [6]. Interestingly, the existence of prion genes and their paralogs appears to be restricted to vertebrates and therefore represents, on the evolutionary timescale, a relatively recent genomic development. Where did the prion founder gene originate from? We recently demonstrated the evolutionary descent of the prion gene from the Zrt-, Irt-like protein (ZIP) family of metal ion transporters [7] and documented that members of the mammalian prion protein family reside in spatial proximity to their ZIP molecular cousins in neuroblastoma cells [8]. More specifically, sequence alignments, structural threading data and multiple additional pieces of evidence placed a ZIP5/ZIP6/ZIP10-like ancestor gene at the root of the PrP gene family (**Table S1**). Amino acid sequence comparisons of the human ZIP proteins argue that ZIP6 and ZIP10, together with their phylogenetically closest paralog ZIP5, constitute a distinct subbranch in this family [9]. What we termed the prion-like (PL) domains of these ZIPs are predicted to form ectodomains that resemble PrP^C^ with regard to orientation and relative distance to their downstream membrane anchorage sites [2]. Within these PL domains, one can readily identify a sequence segment that is characterized by stronger species-to-species sequence conservation than surrounding segments and is flanked by a pair of cysteine residues. These cysteines (which form a disulfide bridge in prion family protein structures) are universally conserved across all known prion or prion-like domains. Throughout this report we will refer to the sequence segment bounded by these cysteines as the cysteine-flanked core (CFC) domain.

ZIP genes date back much further than prion gene sequences. Indeed, related sequences can be found in all kingdoms of life, including bacteria and plants, and the ZIP gene family has undergone independent expansions within the distinct evolutionary lineages. Thus, whereas the genomes of humans and the plant species *Arabidopsis thaliana* code for similar numbers of distinct ZIP proteins (14 and 17 paralogs, respectively), the evolutionary subbranch of the ZIP family with members harboring a prion-like ectodomain underwent a profound expansion only during the early stages of Chordata emergence that was not mirrored in the plant lineage. This development preceded the emergence of the prion gene family and may serve as an explanation for its restricted existence in vertebrates. Today, based on sequence comparisons, four ZIP subbranches can be distinguished. The branch which contains ZIP transporters with a prion-like ectodomain can also be distinguished from other ZIP sequences on the basis of a putative intramembrane metalloprotease signature sequence and is frequently referred to as the LIV-1 subfamily of ZIP zinc transporters (LZTs).

The question arises as to precisely how the prion founder gene was created. Although a number of scenarios regarding the mode of evolution was presented in our original article [7], insights into the mechanistic aspects of the emergence of the prion founder gene based on an in-depth analysis of relevant sequences were lacking. Here we undertook systematic bioinformatic analyses of select prion and ZIP genes to explore whether the mechanism of prion gene evolution can be deduced. We distinguish two genomic rearrangements: (i) the emergence of a first prion-like ectodomain harboring a cysteine-flanked core in a ZIP gene, and (ii) the formation of the prion founder gene. We document that as much as a half-billion years may have separated these two genomic rearrangement events. Surprisingly, our results point to a genomic insertion of processed and reverse-transcribed ZIP-ancestor mRNA as the most parsimonious explanation for the origin of the founder of the prion gene subfamily. We further document that similar insertions involving ZIP transcripts that probably relied on retropositional elements have occurred at other time points in vertebrate evolution.

## Methods

### Multiple sequence alignments

Sequence alignments were carried out using the AlignX feature of Vector NTI Advance 11.0 (Invitrogen, Carlsbad, CA, USA) [10]. A gap opening penalty of 10, gap extension penalty of 0.05 and gap separation penalty range of 8 were utilized in conjunction with the blosum62mt2 score matrix. Local adjustments were made in instances where visual inspection suggested an alternative alignment to the one returned by the algorithm. Sequences were selected for inclusion in this analysis with a view to (i) cover a broad spectrum of organisms ranging from pre-metazoan yeast and choanoflagellates to invertebrates to humans; (ii) depict all mammalian ZIP paralogs that contain a CFC domain; and (iii) represent a broad spectrum of prion sequences from fish to humans. Please see **Figure S1** for a simplified phylogenetic tree that identifies organisms selected for this and subsequent analyses.

### Intron-exon genomic organization

PrP and ZIP genes from a variety of organisms were selected based on their relevance to the ZIP-PrP evolutionary hypothesis [7]. Whenever multiple paralogs of a certain gene were available, the gene with the highest homology (based on protein sequence alignments) to other sequences in the figure was chosen. Intron-exon structures (sequences and information on the lengths of gene segments) and the start and stop codon positions were systematically extracted for each gene of interest from Ensembl (European Molecular Biology Laboratory-European Bioinformatics Institute, EMBL-EBI, and Wellcome Trust Sanger Institute, release 59) and Entrez (National Center for Biotechnology Information, NCBI, GenBank release 180.0) genomic databases (**Table S2**). Transmembrane (TM) region boundaries and CFCs were identified as described previously [7]. The scales of the intron-exon figures were based on the length of the longest gene. The genes were arranged by aligning the 5′ end of their respective CFC domains.

### Synteny analysis

For synteny analyses, the chromosomal locations, lengths and the directionality of the three neighboring genes upstream (5′) and downstream (3′) of prion or ZIP genes of interest were extracted from Ensembl and Entrez genomic databases. To facilitate side-by-side comparisons, genomic regions were depicted with 5′ boundaries of prion or ZIP genes aligned. In instances of uncertain identity (e.g., genes annotated with numerical identifiers), BLAST searches were conducted to establish possible homology relationships amongst genes.

### Pseudogene discovery

Protein sequences of human ZIP6 and ZIP10 were submitted to the PseudoGeneQuest online program (Institute of Medical Technology, Tampere, Finland, version 0.4) [11] to search for known human pseudogenes, pseudogene fragments and interrupted processed pseudogenes. The program used the human genome build 37.1 and known pseudogenes were retrieved from the Pseudogene.org database (version 71). The results were then individually BLAST-searched to determine if the hits indeed constituted ZIP pseudogenes or represented misannotated ZIP paralogs. To identify possible pseudogenes in other organisms, different domains (and combinations thereof) of LIV-1 ZIP sequences from different chordate species, which spanned more than one exon, were BLAST-searched against all genomes available in the NCBI database (GenBank release 180.0) and results showing contiguity in one or more exonic areas were flagged for further analysis. Repetitive elements were identified using the RepeatMasker online interface (Institute for Systems Biology, Seattle, WA, USA, version open-3.2.9) [12].

### Accession numbers

A list of accession numbers for sequences mentioned in this manuscript and the key to species name abbreviations appear in **Table S2**.

## Results

### The cysteine-flanked core within prion-like domains of metazoan ZIP proteins is set apart from surrounding sequences by a high level of positional sequence conservation and a pair of flanking introns

**Figure 1:**
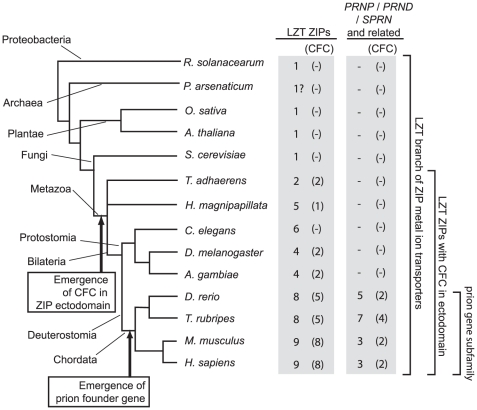
Broad phylogenetic distribution of LIV-1 ZIP metal ion transporters contrasts narrow distribution of prion genes in Chordata lineage. Numbers of LIV-1 ZIP and prion sequences in the selected organisms were extracted from gene data published by the Wellcome Trust Sanger Institute (TreeFam, http://www.treefam.org) and by multiple alignments of ZIP and prion protein sequences. For each organism, the number of the subset of sequences containing a cysteine-flanked core (CFC) domain is indicated in brackets.

**Figure 2:**
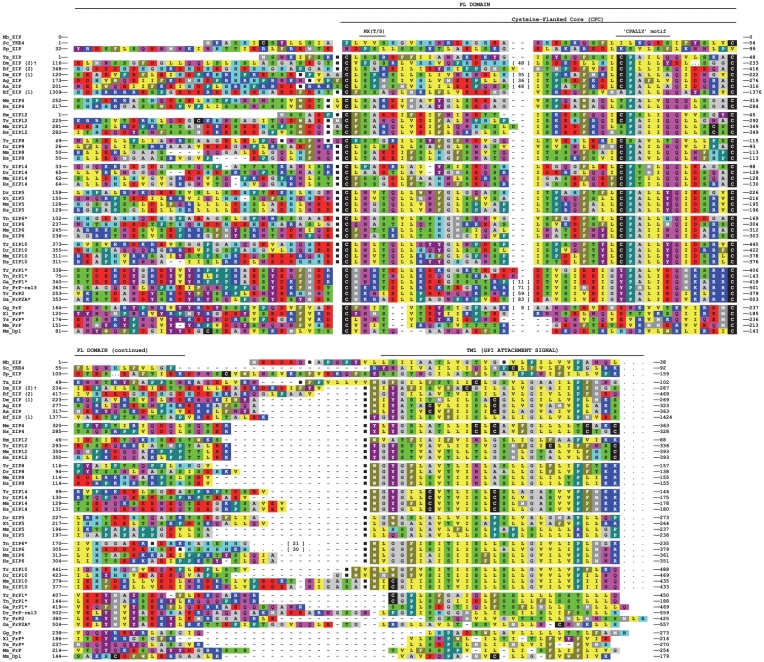
The cysteine-flanked core within the prion-like domain of ZIP proteins is confined to metazoa. Multiple sequence alignment of the prion-like domain of select PrP and ZIP genes from metazoans to mammals. Baker’s yeast (Sc_YKE4), fission yeast (Sp_ZIP) and choanoflagellate (Mb_ZIP) sequences were included in this alignment in the interest of depicting a small number of representative LZT protein sequences outside of the metazoa realm. Their ectodomains appear, however, to lack a CFC domain based on (i) poor alignment, (ii) the absence of a ‘CPALLY’ motif and (iii) the absence of conserved introns. Black squares (▪) indicate the position of introns and asterisks (*) denote sequences for which complete intron/exon annotations were not available. Numbers in square brackets ([X]) indicate the length of a stretch of amino acids omitted for the purpose of clarity in a specific section of the alignment. Please note that Dm_ZIP (2) (marked with dagger symbol †) is the same protein sequence as that encoded by the *Drosophila melanogaster fear-of-intimacy* (*foi*) gene. Please see **Table S2** for a complete list of scientific and common names of species referred to in this alignment with two-letter abbreviations.

Our previous analyses revealed the existence of a PL domain in a subset of genes belonging to the LIV-1 subfamily of ZIP zinc transporters (LZT) in diverse non-vertebrate organisms for which complete genomic data were available at the time, including *D. melanogaster* (fruitfly) and *H. magnipapillata* (jellyfish) [7]. The ongoing international genome sequencing activities have in recent times generated additional genome depositories for a range of organisms with more primitive body plans. Thus, to refine the evolutionary time point at which the first CFC domain may have emerged in a ZIP ancestor, we extended our search to the genomes of fungi, other relevant unicellular eukaryotes and early metazoa. These genomic queries made use of the PSI-BLAST algorithm and interrogated the respective genomic databases with sequence templates that forced perfect matching in highly conserved sequence positions (derived from a multiple alignment of prion-like domains we had identified in ZIP proteins earlier) but allowed variation in other positions of the sequence. This approach failed to detect ZIP gene sequences with a predicted prion-like domain in all genomes of unicellular organisms we investigated but revealed the existence of a ZIP sequence with a characteristic CFC domain in *Trichoplax adhaerens* (Ta) (**Figure 1** and **Table S2**). A multiple alignment of prion-like domains of ZIP gene sequences from diverse organisms (please see **Figure S1** for a summary of species used in this and subsequent analyses), including Ta, and prion genes revealed a dichotomy in the degree of sequence conservation within the globular PL domain itself, i.e., sequences N-terminal to the CFC domain are conspicuously enriched in charged residues but show, in contrast to sequences within the CFC, relatively little positional conservation (**Figure 2**). Interestingly, highly conserved intron-exon boundaries can be found immediately N-terminal and in close C-terminal proximity to the CFC not only in all human LZTs (LIV-1 ZIP zinc transporters), which contain this domain, but also in distant LZT sequences found in the genomes of species that range from *Trichoplax* to fruitfly to pufferfish. Consistent with their ancient origins, the lengths of these positionally-conserved introns are known or predicted (in instances where no transcripts are available) to vary widely from a few nucleotides to thousands of base pairs. This analysis further revealed that ZIP zinc transporter genes of unicellular organisms neither code for a CFC nor feature introns in the respective segments of their genes. In fact, their protein sequences do not align N-terminal of the transmembrane domain and were merely included in this analysis to document these observations.

### ZIP genes of all evolutionary lineages are characterized by complex intron-exon structures not observed in prion gene sequences

**Figure 3:**
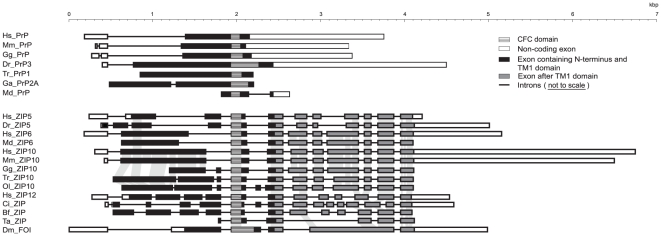
Multiple introns observed in the coding regions of ZIP genes are missing from prion genes. Complex intron/exon arrangements of ZIP genes contrast the genomic organization of prion genes characterized by a coding sequence that is confined to one or, in rare instances, two exons. Only exons are depicted to scale. Black, hatched, grey or white fillings depict exons coding for the ectodomain, the cysteine-flanked core (CFC), the C-terminal multi-spanning transmembrane domain of ZIP transporters or non-coding segments of a given transcript, respectively. The black solid lines connecting exon boxes indicate introns. Untranslated regions (UTRs) are not depicted for a subset of sequences lacking reliable relevant annotation in the databases. For Tr_PrP1, part of the 5′ UTR, the ORF and the 3′ UTR are encoded in a single exon. Dm_FOI represents the ZIP ortholog in *D. melanogaster* with strong sequence similarity to mammalian ZIPs 5, 6 and 10. kbp, kilobase pairs.

The comparison of transcript structures of a set of related genes can sometimes shed light on the evolutionary history that links them to a common ancestor [13]. In particular, the number of exons and the relative position of intron-exon boundaries in relevant orthologous sequences can provide the basis for forming hypotheses regarding evolutionary relationships. To that end, we expanded upon the initial determination of introns flanking the CFC domain and investigated the intron-exon structure of the coding sequences of prion genes in vertebrates and of a representative subset of ZIP genes from diverse organisms (**Figure 3**). Species included in these analyses were selected with a view to (i) capture distant branches of the evolutionary tree, (ii) include PrP and ZIP gene sequences that are most similar (e.g. from pufferfish) or relatively distantly-related to each other (e.g. human sequences) according to our previous ZIP-prion evolutionary analyses [7], and (iii) extend the analysis of ZIP sequences to genomic lineages whose divergence predates the split of PrP and ZIP sequences and, thus, may be meaningful for deducing the gene structure of ZIP genes at the time when the prion gene emerged (**Figure S1**). Analyses relied on intron-exon genomic annotations provided by Ensembl and Entrez databases. Whenever annotations were ambiguous or conflicting, clarification was sought by comparing expressed sequence tag (EST) entries to the corresponding genomic sequences. In-depth analyses of prion genes in diverse organisms which preceded this work have repeatedly revealed a common gene structure composed of one or two short 5′ noncoding exons and a relatively long exon that codes for a short 5′ untranslated region (UTR), the entire open reading frame (ORF) and a 3′ untranslated region [14], [15], [16], [17], [18]. Thus, the emphasis in this analysis was not on prion genes but on ZIP genes for which no detailed analyses had been undertaken.

Even at cursory inspection, the results revealed strikingly different intron-exon gene organizations of prion and ZIP genes. Consistent with data from the aforementioned studies, the ORFs of almost all vertebrate prion genes were confirmed to be contained in single exons. Exceptions to this genomic organization represent the prion gene homologs in stickleback and opossum, which apparently underwent genomic rearrangements that caused the coding sequences to be split into two exons. In contrast, the ORFs of all vertebrate ZIP genes we analyzed predict the splice-removal of multiple introns for generating the respective messenger RNAs. A closer look at ZIP genes revealed two categories of introns: (i) introns which display low positional conservation even amongst closely related members of the family, and (ii) introns that are highly conserved. For examples, two highly conserved introns which flank the CFC domain were observed in all LZT ZIPs that contain a prion-like ectodomain. In contrast to intron positions, intron lengths are known to change relatively rapidly in evolutionary time and are therefore a poor indicator of sequence relationships. Consistent with this general observation, ZIP genes included in this analysis display a remarkable variation in the lengths of their corresponding introns, with human ZIP5 and ZIP12 serving as a pair of genes exhibiting multiple relatively short and long positionally-conserved introns, respectively (**Figure S2**). Significantly, the absence of introns flanking the CFC region in today's prion sequences suggests that these introns disappeared shortly after or during the emergence of the prion gene founder from its ZIP ancestor.

### No shared genes in the genomic neighborhoods of ZIP and prion genes

**Figure 4:**
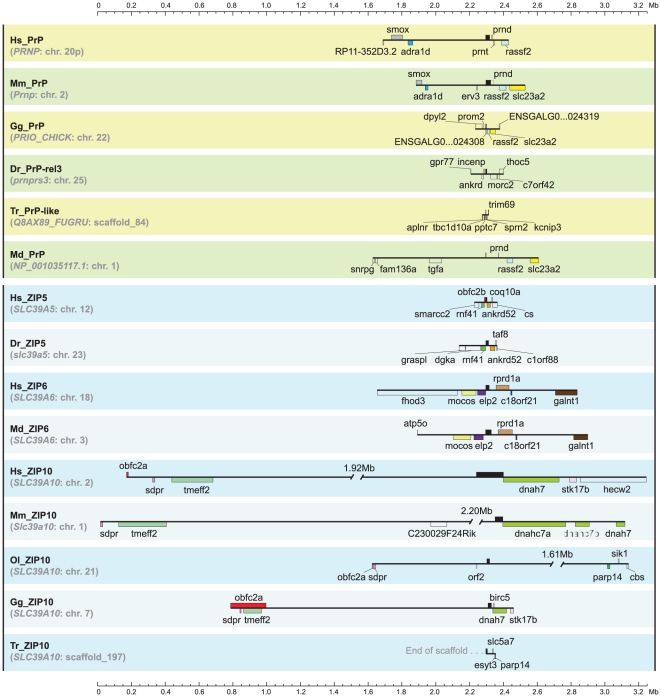
Lack of shared genes in proximity of PrP and ZIP genes. The synteny analysis was restricted to three adjacent genes on either side of the relevant PrP and ZIP genes. Synteny was determined to be restricted to ortholog sequences, with paralogs of the gene *obfc2* observed adjacent to both ZIP5 and ZIP10 genes serving as notable exceptions. Black boxes depict the genes of interests (PrP/ZIP), and colored boxes represent proximal genes. Please note that Tr_ZIP10 maps to the 5′ boundary of a genomic contig for which the adjacent genomic segment is not annotated. Black solid lines indicate interspersed non-coding regions. Mb, megabase pairs.

Synteny analyses can be a powerful vehicle not only for establishing gene homology relationships, but also with respect to providing clues about the mechanistic origins of new genes. We therefore conducted an analysis of the genetic neighborhoods of select prion and ZIP genes. Specifically, the identity and relative genomic position of the three genes which map to genomic regions immediately adjacent to either side (5′ versus 3′) of the selected ZIP and PrP genes were recorded using Ensembl and Entrez genomic databases. In instances where the gene nomenclature did not readily reveal the identity of a gene, BLAST searches were conducted to establish possible relationships to other genes recorded in this manner. Consistent with previous reports, organisms as distant to each other as pufferfish and humans exhibit synteny on the 3′ side of prion genes where *prnd* orthologs, *rassf2* and *slc23a2* genes are shared [5], [19]. Similarly, strong syntenic relations among ZIP gene orthologs are easily detected in all vertebrate sequences we scrutinized. For example, ZIP5 was flanked by *rnf41* and *ankrd52*, and ZIP6 showed synteny with *mocos*, *elp2*, *rprd1a*, *c18orf21* and *galnt1* (**Figure 4**). The genes *tmeff2*, *sdpr* and *stk17b* were within the physical proximity of ZIP10 in human, chicken and fish genomes. More importantly, evidence for synteny could even be obtained for ZIP paralogs. Namely, the homologous variants of the *obfc2b/a* gene were detected in close proximity to human ZIP5 and ZIP10 genes, probably indicating an evolutionarily conserved linkage to the region that once hosted an ancestor of the subbranch of ZIP genes to which ZIP5 and ZIP10 belong. Notably though, no gene homologous to *obfc2b/a* was detected in proximity to ZIP6 genes, the third paralog in this ZIP subbranch, or the prion protein gene. And whereas two genes belonging to the ankyrin gene superfamily were located in spatial proximity to both zebrafish PrP (*ankrd*) and ZIP5 sequences from various organisms (*ankrd52*), a closer comparison of relevant sequences failed to reveal orthologous relationships for these genes and instead suggested *ankrd* and *ankrd52* to be distant members of a large and diverse gene family.

Taken together, no evidence for shared genomic context in proximity to PrP and ZIP genes emerged from this analysis, corroborating the impression that the homology of prion and ZIP genes may not extend beyond their respective coding regions.

### Pseudogene analyses uncover instances of genomic insertions of spliced and reverse-transcribed ZIP transcripts in vertebrates

The data presented thus far suggested the intriguing possibility that a spliced and reverse-transcribed ZIP transcript may have served as an intermediate during the generation of the prion founder gene. We therefore wondered whether instances of retroposition of all or parts of a ZIP transcript harboring a prion-like domain can be traced in current genomes. We initially restricted our search to the human genome. A query of the PseudoGeneQuest online tool [11] with the human ZIP6 sequence returned multiple hits of which 10 were designated by the program as pseudogenes, 1 as a pseudogene fragment and 2 as interrupted processed pseudogenes. Similarly, 9 sequences which were flagged as pseudogenes, 2 as pseudogene fragments and 2 as interrupted processed pseudogenes were returned by the algorithm when queried with a human ZIP10 sequence (interrupted processed pseudogenes are search results possessing repeat content which is >50% of the length of the target) [20]. A subsequent closer analysis of these hits based on BLAST searches identified most of them as ZIP paralogs (of which there are 14 in the human genome) which had been misinterpreted by the algorithm to represent candidate pseudogenes. However, one interrupted processed pseudogene returned for both the ZIP6 and ZIP10 queries was confirmed by us to represent a *bona fide* ZIP pseudogene located on human chromosome 1 (residues 48,061 to 49,786, clone RP11-365D9, locus AL583844.11). In fact, the genomic Entrez/NCBI annotation of this clone already identified this sequence as a ZIP14 pseudogene. Alignment of this region with the human ZIP14 parent gene located on the short arm of chromosome 8 further refined the boundaries of the retroposed segment and revealed that the entire coding sequence of the ZIP14 parent gene is retained in this pseudogene sequence, but considerable sequence decay has accumulated since its formation (**Figure S3A**). A recent update to the human Ensembl/Vertebrate Genome Annotation (VEGA) genome database indicated the existence of a second ZIP pseudogene in humans. This pseudogene is located on chromosome 22 and derived from the ZIP1 parent gene (**Figure S3B**). However, given that ZIP1 does not contain a PL domain in its sequence, this pseudogene is of lesser relevance in the context investigated here.

**Figure 5:**
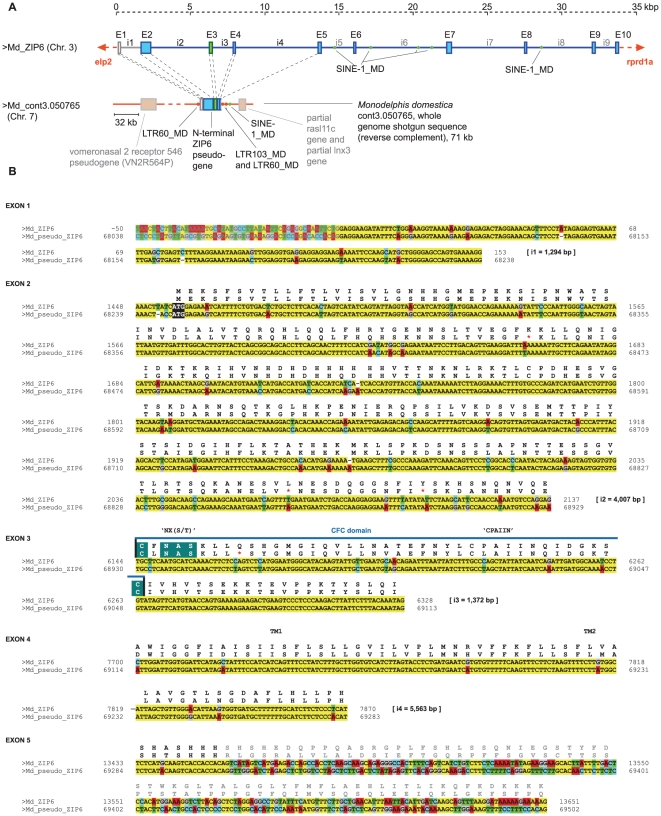
Evidence for a C-terminally truncated ZIP6 pseudogene in the opossum genome. **A**. An N-terminal ZIP6-like pseudogene was identified on chromosome 7 of *Monodelphis domestica* (cont3.050765, GenBank: AAFR03050766.1), which consisted of exons 2, 3, 4 and parts of exons 1 and 5. Exon 3 which codes for the CFC is depicted in light green color. Long terminal repeats (LTRs) and short interspersed nuclear elements (SINEs) in the vicinity of the pseudogene are marked. Please note that short direct repeats immediately flanking the pseudogene were not detected. Similarly, no evidence of a poly-A tail could be observed, consistent with the retroinsertion of a C-terminally truncated ZIP6 transcription product. **B**. Sequence alignment of opossum ZIP6 with the pseudogene and its flanking sequences, clearly demarcating the boundaries of retroinsertion. Identical base pairs in the two sequences are highlighted in yellow, and sequence features such as the CFC domain are marked at the amino acid level. kbp, kilobase pairs.

We hypothesized that the development of the prion founder gene might have been accompanied by a loss of most of the C-terminal domain of its ancestral ZIP parent gene. Assuming that retroposition might have been the mechanism, we wondered whether such an event was a unique occurrence or whether it would be possible to find evidence that a similar ZIP retroposition paralleled by the loss of C-terminal transmembrane domains also occurred at a different time. To address this question, we next searched the genomes of available chordate organisms for LIV-1 ZIP-like sequences that (i) were contiguous at conserved exon boundaries within segments of ZIP genes that code for their prion-like domain, and (ii) did not align to stretches of conserved C-terminal ZIP sequences. The objective was not to generate an exhaustive list of candidate sequences but to determine whether at least one such sequence could be found. Indeed, on chromosome 7 of the gray short-tailed opossum (*Monodelphis domestica*) a sequence was found which matched the filtering criteria (**Figure 5**). The respective pseudogene was flanked by a number of repetitive elements (**Figure 5A**) and aligned to exons 2, 3, 4 and parts of exons 1 and 5 of the opossum ZIP6 gene which maps to chromosome 3 (**Figure 5B**). This pseudogene features four stop codons but otherwise has experienced a low degree of sequence decay. Taken together, the pseudogene analyses uncovered specific examples of independent insertion events of spliced and reverse-transcribed ZIP transcripts in present-day vertebrate genomes.

## Discussion

The recently-proposed ZIP-prion evolutionary link [7] raised the possibility that a close examination of relevant genomic sequences may reveal insights into genomic rearrangements which precipitated the emergence of prion genes in the vertebrate lineage. In the following paragraphs we will present an argument based on the data from this report which proposes that the emergence of the prion founder gene depended on two genomic rearrangements which occurred hundreds of millions of years apart.

**Figure 6:**
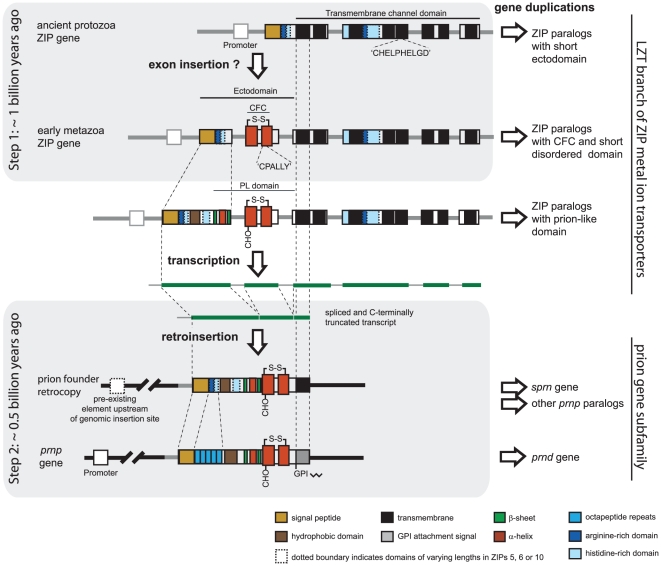
Two-step model of emergence of prion gene from a ZIP ancestor. At the time in evolution when early metazoa emerged, a CFC domain was inserted into an ancient ZIP transporter or evolved *de novo*. During early vertebrate speciation, a descendant of this ZIP ancestor, with ectodomain features resembling present-day ZIPs 5, 6 and 10, gave rise to a processed transcript which was reverse-transcribed and inserted into a genomic region that shares no synteny relationship with the parent gene. Through acquisition of a nearby 5′ promoter element, this retrocopy may have evolved into a fully functional retrogene – the first prion gene. An additional expansion of the subfamilies of LZT and prion genes occurred through gene duplication events. Genomic elements in this figure are not drawn to scale. The depiction of intron positions for the ZIP gene are based on the intron-exon structure of the *Trichoplax adhaerens* LIV-1 ZIP gene harboring a CFC domain described in this manuscript.

We will discuss that the first of these two events on the path to the prion founder gene may have involved the insertion of a CFC domain into a preexisting ZIP ancestor. This event likely occurred around the time when multicellular mobile metazoa emerged on the planet, possibly more than a billion years ago [21]. The second event, i.e., the actual formation of the prion founder gene, can be traced back to a time before the divergence of teleosts and tetrapods, approximately a half-billion years ago (**Figure 6**). The proverbial ‘smoking gun’ which would simplify the reconstruction of this genomic rearrangement (e.g., flanking short direct repeats and/or the presence of remnants of a poly-A tail) may no longer exist in the genomes of contemporary vertebrate species. Nonetheless, the cumulative data we presented relate a consistent story and suggest that the formation of the prion founder gene may have involved the genomic insertion of a reverse-transcribed ZIP transcript.

### Emergence of prion-like ZIP ectodomain in early metazoa

Data presented in this manuscript established that ZIP sequences containing CFC domains can be identified in the genomes of metazoa with relatively primitive body plans, including the amoeba-like organism *Trichoplax adhaerens* (Ta) and cnidarians, but these domains seem to be absent in ZIP genes of all other branches of life. Thus, around the time when the metazoa lineage emerged, the CFC domain may have either gradually evolved or become inserted as a module into a preexisting ZIP gene (**Figure 1**). Multiple alignments of prion and ZIP sequences from a diverse selection of organisms undertaken for this work revealed a dichotomy in the degree of sequence conservation within the globular PL domain itself, i.e., sequences flanking the CFC domain are conspicuously enriched in charged residues but, in contrast to sequences within the CFC, show relatively little positional conservation (**Figure 2**). A number of alternative (and not necessarily mutually exclusive) explanations come to mind that may have limited the ability of the CFC to diversify: (i) the currently unknown function or molecular interactions of the CFC might have placed limitations on sequence variation; (ii) the predicted existence of a disulfide bridge formed between cysteine residues (so far only proven to exist in the CFC of tetrapod PrP^C^ and Dpl) at its boundary may constitute a structural constraint that restricted sequence evolution; and/or (iii) a different rate of evolution might be the consequence of a genomic organization that causes this genome segment to evolve at a different pace than the surrounding sequences. Indeed, conserved exon/intron boundaries can be found immediately N-terminal and in close proximity to the C-terminal boundary of the CFC domain in the genomes of species ranging from *Trichoplax* to humans. It is therefore likely that the emergence of this domain was based on exon shuffling or an exonization of a preexisting intron, a process which can, for example, be triggered by intronic insertion of a retroelement providing novel splice acceptor motifs [22], [23]. Consistent with their ancient origins, the lengths of the positionally-conserved introns flanking the CFC vary widely from a few nucleotides to thousands of base pairs in LZT genes. The alternative model based on which the CFC domain was generated through gradual sequence evolution is less appealing because it fails to explain the concomitant emergence of the two highly-conserved flanking introns.

### Generation of prion founder gene in vertebrates

The absence of introns flanking the CFC region in today's prion sequences (**Figure 3**) suggests that these introns disappeared shortly after or during the emergence of the prion gene founder from its ZIP ancestor. While these introns may indeed have disappeared after the actual gene duplication event and independent of it, this explanation neither represents the most parsimonious model nor does it suggest a satisfying answer for why intron loss in these positions occurred in the prion gene founder but is not observed in CFC-containing ZIP gene sequences. The literature surrounding intron loss and gain seems contradictory: on the one hand, forces of genome miniaturization have given rise to massive-scale intron loss in individual species [24], and on the other hand, intron loss has been described as a rare event relative to other types of genomic rearrangements [25], [26], a quality exploited in studies that use comparative intron mapping to determine deep evolutionary histories of gene families [27], [28]. A large-scale comparison of mouse and human genomes revealed, for example, that introns in these two species are only changed in 0.08% of positions, indicating a more than 1,000-fold higher level of conservation when compared with protein sequence changes [29]. Whenever intron loss is observed for two adjacent positionally-conserved introns, it appears to be the result of a reverse transcription of RNA intermediates [25]. Mechanistically, RNA intermediates play a role in two types of intron loss events: gene conversions by recombination with spliced transcripts from the affected gene, and retroposon-mediated gene transfers [26], [30]. Whereas the former mechanism converts the gene in its original genomic environment, the latter causes a transposition of a spliced copy of the original gene into a distant genomic acceptor site, generally assumed to represent transcriptionally active and open chromatin [31], [32]. Consequently, an important criterion for the designation of retrocopies is the loss of at least two positionally-conserved introns in regions that can be aligned to homologous parent genes [33], [34]. In the context discussed here, the application of this criterion suggests that the emergence of the prion gene founder may have been the result of an ancient germline retroposition event. The truncation of the prion gene founder could then have been the consequence of a frequently-observed shortening of mRNA sequences before or during reverse transcription [35], or could have occurred following the genomic insertion but prior to the divergence of PrP sequences as a result of speciation.

Following their genomic insertion, the majority of retrocopies turn into pseudogenes by falling transcriptionally silent and being subjected to relatively rapid genomic mutations, insertions and deletions that lead to sequence decay and can eventually cause the elimination of pseudogene sequences [36]. These rapid evolutionary changes to pseudogene sequences occur because regulatory elements that could drive their expression and, consequently, help to realize a stabilizing selective advantage, are missing. Thus, for sustained survival of a retrocopy, it is critical that the genomic insertion event occurs in the vicinity of a preexisting promoter or proto-promoter that can be hijacked for transcriptional activity [30], [34]. When genomic insertions do not occur immediately proximal to a preexisting promoter but in some 3′ distance to it, retrocopies can adapt 5′ sequences and even untranslated exon/intron structures for gene regulatory purposes on their way to become transcriptionally-active retrogenes [37]. Thus, the existence of noncoding exons and introns in the 5′ untranslated region of today's prion genes is not inconsistent with this model but represents a frequent occurrence in retrogenes [38]. Alternatively, retrogenes have been shown to acquire exons *de novo* during evolution. For example, a study of >1,000 retrocopies in the human genome revealed a surprisingly large percentage of retrogenes (27 out of a total of 120 retrocopies which had developed into *bona fide* genes) that had acquired untranslated exons in this manner [34]. In the absence of strong sequence conservation, the effects of genomic rearrangements and divergent sequence evolution which accumulate in a given pair of retro- and parent genes over time may mask the ability to recognize the origins of the former. For example, the gene encoding HNRPF, a protein involved in RNA processing, was not recognized as a retrogene until recently, possibly because it recruited three 5′ untranslated exons [34]. The most conspicuous lingering characteristic indicating retrogene origins might be the absence of introns within ORFs, a school of thought that provoked the proposition that many of the approximately 15% of genes in the human genome lacking introns in their ORF may have arisen by retroposition [39].

Because retroposition is accompanied by the loss of the surrounding genomic sequences, the absence of homology of promoter sequences and synteny relationships of a retrocopy and its parent gene constitute additional criteria routinely used for the distinction of retrocopies from segmentally duplicated genes.

However, because of the extensive evolutionary time which has passed since the emergence of the prion founder gene and given the relatively rapid diversification of non-coding sequences, a comparison of promoter sequences seemed futile as no sequence conservation would be expected at this time, regardless of the mechanism of evolution. A promoter comparison we undertook for other purposes confirmed this prediction but was not included to avoid distraction from the more meaningful analyses.

In the case of prion gene family members, previous synteny analyses not only provided a framework for comparing the evolutionary links amongst prion-related genes, but also led to an intriguing model which posits that all prion genes known to date have emerged from a common prion gene founder [40]. The examinations of genetic neighborhoods undertaken in this work revealed robust synteny within ortholog comparisons of different ZIPs or prion genes (**Figure 4**), but failed to detect synteny across paralog boundaries with one notable exception: the gene *obfc2b/a* was found to be shared in proximity of both ZIP5 and ZIP10 genes of the ZIP LIV-1 subfamily. This is relevant as comparative genomic analyses we have undertaken suggest that the subbranch of the ZIP gene family populated by ZIPs 5, 6 and 10 may have undergone an expansion around the time when the prion founder gene emerged [7]. This is evident based on the existence of ZIPs 5, 6 and 10 genes in some teleost and tetrapod genomes but not in the early chordates. Thus, the synteny across ZIP5-ZIP10 paralog boundaries served as a positive control in this analysis. It documented that despite the approximately half-billion years which have passed since the divergence of the paralogous pair, this event can still be identified to have been mechanistically based on a duplication of a genomic segment containing a predecessor ZIP gene and its adjacent genes. Thus, it is conceivable that synteny between prion and ZIP genes could be observed in contemporary genomes if the emergence of the prion founder gene had relied on a similar genomic duplication event.

An important aspect in prion pathobiology which also relates to the emergence of the prion founder gene is the evolutionary time point at which the protein became capable of infection and aggregation. However, given that very little is known about prion disease outside of the mammalian clade, the characterization of the evolution of prion infectivity requires further research.

### Other retropositional events

Just as inductive reasoning draws strength from specific repeated observations, the notion of retrocopy origins of the prion founder gene would be easier to embrace if other instances of retrocopy events involving ZIP genes could be traced in existing genomes. A non-exhaustive search in genomic databases we report in this manuscript revealed that independent retroinsertions of ZIP transcripts containing prion-like ectodomains have indeed occurred in the opossum and human genomes (**Figures 5** and **S3**). Incidentally, the opossum ZIP pseudogene was derived from a ZIP6 parent gene, a member of the very subbranch of ZIP family genes which we proposed to have given rise to the prion founder gene [7]. Remarkably, a comparison of gene boundaries of the pair of opossum ZIP6 retrocopy and parent gene revealed that the retrocopy lacks most of the C-terminal sequences coding for the multi-spanning transmembrane domain of its parent gene. Thus, the opossum with its ZIP6 pseudogene can be viewed as demonstrating a re-enactment of the ancient genomic rearrangement which may have caused the loss of C-terminal domains of the prion founder gene.

**Figure 7:**
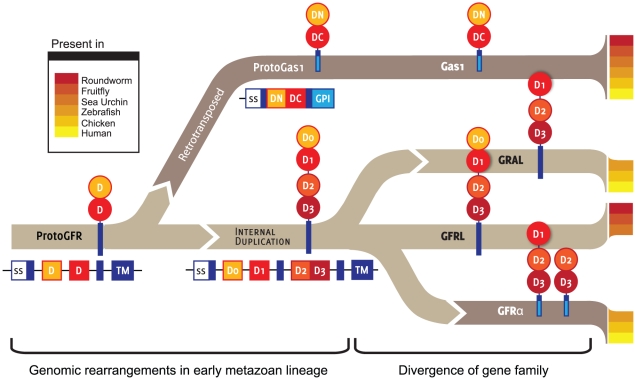
Precedent of retroposition event leading to a subbranch of GPI-anchored proteins within family of transmembrane proteins. Schematic representation of proposed mode of evolution of glial cell-derived neurotrophic factor (GDNF) family ligand receptor (GFRα) members. Horizontal and vertical cartoons depict modular gene and protein organization of GFRα members, respectively. Please note both the absence of introns and the emergence of the GPI membrane attachment mode in the Gas1 subbranch of GFRα proteins following a retroposition event which occurred early during metazoan speciation.

Is there a precedent of an unrelated gene family in which a phylogenetic subbranch originated from an ancient retroinsertion event? The gene family of glial cell-derived neurotrophic factor (GDNF) family ligand receptors (GFRα) may serve as an example: within the *GFRA* gene family, a majority of sequences share a common exon-intron structure. However, growth arrest-specific 1 (*GAS1*) genes – members of a *GFRA* gene family subbranch expressed in species as diverse as roundworms, honey bees and humans [41], [42] – lack all introns and have been proposed to have originated from an ancient retroinsertion event [43], [44] (**Figure 7**). Intriguingly, the parallels do not end there, as GAS1 (like the prion protein) is a glycosylphosphatidylinositol (GPI)-anchored member in a protein family that contains both transmembrane (e.g., GDNF family receptor alpha-like, GFRAL) and GPI-anchored proteins (GFRA 1-4) [42]. Thus, in both the GFRα family and the ZIP superfamily, a retrotransposition event may have given rise to a subbranch of C-terminally truncated genes. These observations are consistent with previous reports by other groups which established that a mere C-terminal truncation of genes coding for transmembrane proteins at a site adjacent to transmembrane-coding sequences can be sufficient to generate a signal sequence for the attachment of a GPI anchor [7], [45].

Taken together, our bioinformatic analyses of prion and ZIP genes and their genetic environments suggest that retroposition was the likely mode of emergence of prions from a LIV-1 ZIP ancestor molecule. It is anticipated that this model can be further refined once additional genome sequences of species with relevance for elucidating pre-vertebrate evolution become available. Potentially more rewarding, however, might be to uncover (i) where the CFC domain within metazoan ZIP transporters originated from, and (ii) whether any molecular cousins of the prion protein exist which descended from the independent retroposition of ectodomain-coding sequences of ZIP transporters lacking a CFC domain.

## Supporting Information

Figure S1 **Tree diagram depicting species utilized for genomic analyses in this study.** In all instances, species included for a given analysis were to provide a broad and most informative sample and, at the same time, minimize redundancy. Because the questions which were addressed differed from analysis to analysis, the most relevant sample of gene sequences differed accordingly. MA, multiple alignment; EI, exon-intron; SA, synteny analysis.
(PDF)

Figure S2 **Multiple introns observed in the coding regions of ZIP genes are missing from prion genes.** Alternative presentation of data from intron/exon analysis shown in Figure 3 with both intron and exon lengths depicted to scale.
(PDF)

Figure S3 **Evidence for the existence of ZIP pseudogenes in the human genome.** A. Human chromosome 1 contains a retrocopy of the human ZIP14 gene coded within the long arm of chromosome 8. The retrocopy is embedded within a relatively long intron of the guanine nucleotide binding protein gamma 4 (*GNG4*) gene. It exhibits telltale signs of sequence decay associated with pseudogenes such as an accumulation of multiple translation stop codons and the presence of more than a dozen predicted frameshifts relative to the predicted mRNA sequence of its parent ZIP14 gene. B. A relatively short ZIP1 pseudogene corresponding to a C-terminal segment of its ZIP1 parent gene coded within chromosome 1 can be identified on human chromosome 22. The pseudogene sequence features a translation stop codon and two predicted frameshifts.
(PDF)

Table S1 **Summary of evidences presented in support of evolutionary descent of PrP gene family from ZIP metal ion transport ancestor gene.**
(PDF)

Table S2 **Protein accession numbers and abbreviations of species names.**
(PDF)
