# Supplementary figures and images for: Evidence for Retrogene Origins of the Prion Gene Family
Source: PLoS One. 2011 Oct 27;6(10):e26800. doi: 10.1371/journal.pone.0026800 (PMC3203146; doi:10.1371/journal.pone.0026800)

Figure S1

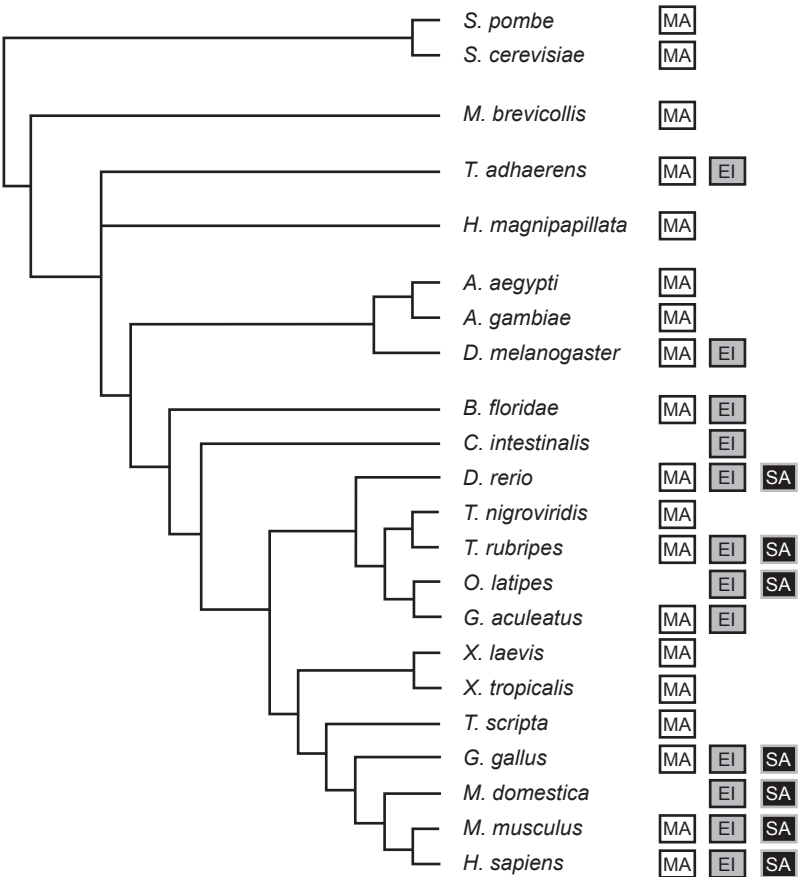

Supplement: Figure S1 — Tree diagram depicting species utilized for genomic analyses in this study. In all instances, species included for a given analysis were to provide a broad and most informative sample and, at the same time, minimize redundancy. Because the questions which were addressed differed from analysis to analysis, the most relevant sample of gene sequences differed accordingly. MA, multiple alignment; EI, exon-intron; SA, synteny analysis. (PDF) [file pone.0026800.s001.pdf]

Figure S2

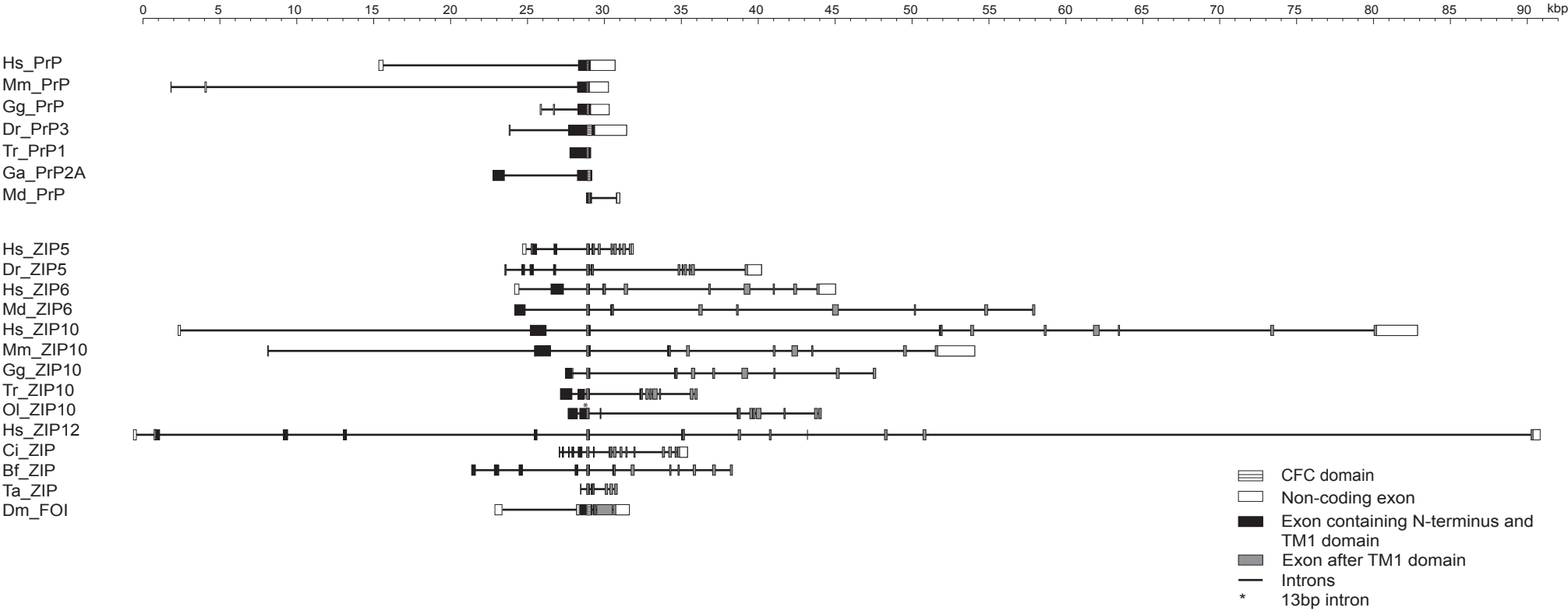

Supplement: Figure S2 — Multiple introns observed in the coding regions of ZIP genes are missing from prion genes. Alternative presentation of data from intron/exon analysis shown in Figure 3 with both intron and exon lengths depicted to scale. (PDF) [file pone.0026800.s002.pdf]

Figure S3

A

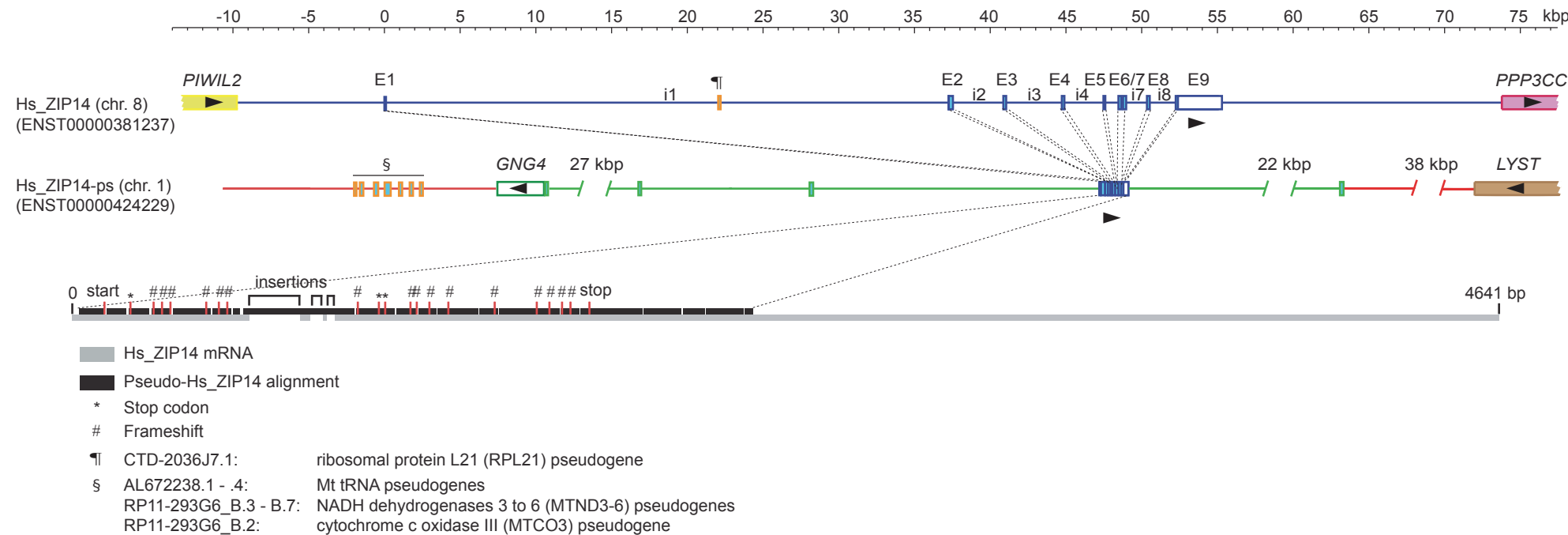

B

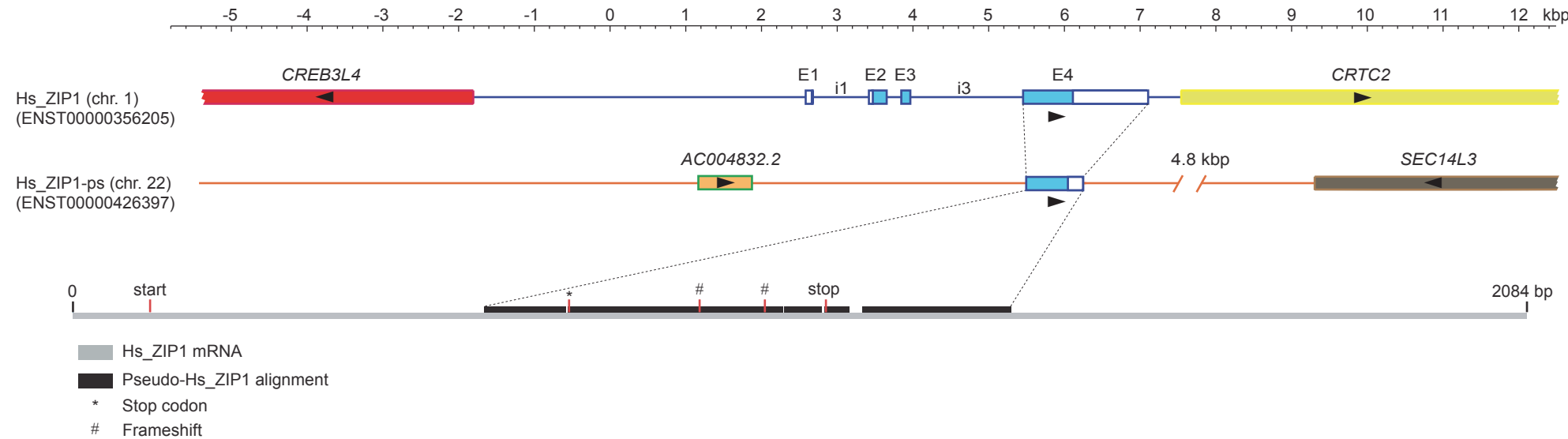

Supplement: Figure S3 — Evidence for the existence of ZIP pseudogenes in the human genome. A. Human chromosome 1 contains a retrocopy of the human ZIP14 gene coded within the long arm of chromosome 8. The retrocopy is embedded within a relatively long intron of the guanine nucleotide binding protein gamma 4 (GNG4) gene. It exhibits telltale signs of sequence decay associated with pseudogenes such as an accumulation of multiple translation stop codons and the presence of more than a dozen predicted frameshifts relative to the predicted mRNA sequence of its parent ZIP14 gene. B. A relatively short ZIP1 pseudogene corresponding to a C-terminal segment of its ZIP1 parent gene coded within chromosome 1 can be identified on human chromosome 22. The pseudogene sequence features a translation stop codon and two predicted frameshifts. (PDF) [file pone.0026800.s003.pdf]
