# Supplementary material for: Evidence for Retrogene Origins of the Prion Gene Family
Source: PLoS One. 2011 Oct 27;6(10):e26800. doi: 10.1371/journal.pone.0026800 (PMC3203146; doi:10.1371/journal.pone.0026800)
Supplement: Table S1 — Summary of evidences presented in support of evolutionary descent of PrP gene family from ZIP metal ion transport ancestor gene. (PDF) [file pone.0026800.s004.pdf]

Table S1. Summary of evidences presented in support of evolutionary descent of PrP gene family from ZIP metal ion transport ancestor gene.

Evidence Category		Description of Evidences
1. Sequence	a.	ZIP10 constitutes the only non-prion gene hit by SCOP "prion-like" HMM (out of 120,000 entries in LOCATE human-mouse protein database).
	b.	COMPASS profile-profile analysis passed homology E-value threshold and confirmed that similarity of PL domain sequences is not merely restricted to spurious outliers.
	c.	The GPI-attachment sequence of prion gene sequences shows sequence similarity with the TM1 domain found in ZIPs. Precedents exist for the transformation of a transmembrane sequence into a signal peptide for GPI anchor attachment.
	d.	An additional pair of ZIP and prion gene sequences (zebrafish ZIP5 / pufferfish Sho2) exhibits a degree of sequence identity/similarity which falls on the significance threshold indicating homology.
	e.	ZIP genes contain histidine-rich repeat motifs reminiscent of octarepeats in prion sequences.
	f.	A zebrafish PrP sequence has been documented which shares the presence of N-terminal $[HX]_n$ clusters with ZIPs.
2. Structure	a.	A common distance of cysteine-flanked core domains to membrane attachment sites is observed in both prion and ZIP protein families.
	b.	Precedents of protein families exist with individual members employing transmembrane domains or GPI anchors for membrane attachment.
	c.	A systematic attempt to thread ZIPs 5/6/10 to any protein structure in the PDB led to the independent assignment of the prion fold.
	d.	The prion protein structures are the only fold templates onto which ZIP sequences can be threaded with scores that pass the threshold for significant homology.
	e.	ZIPs 5/6/10 are expected to display dichotomy of disordered N-terminal sequences and globular PL domains, reminiscent of prion proteins.
3. Function	a.	Consistent with multiple lines of evidence suggesting that proteins harboring the prion fold can bind to each other, ZIP proteins co-purified with prion proteins in this study.
	b.	While many proteins are known to bind divalent cations, PrP and ZIPs belong to a small group of proteins known to capture divalent cations at multiple binding sites embedded within disordered extracellular domains.
	c.	Both the prion protein and ZIPs 5/6/10 have been shown to transport zinc ions across the plasma membrane.
	d.	ZIP6 and PrP knockouts have been shown to display a rare common phenotype in zebrafish (inhibition of gastrulation / altered E-cadherin expression).
4. Localization	a.	ZIPs 5/6/10 and prion proteins share localization to the plasma membrane.
	b.	ZIPs 5/6/10 display common orientation of shared sequence motifs with regard to the plasma membrane.
	c.	Predominant tissues of expression of ZIPs 5/6/10 are reminiscent of PrP/Sho/Dpl gene expression profiles.
5. Phylogenetics	a.	Comparison of orthologous ZIP and prion sequences indicates divergent sequence evolution consistent with phylogenetic relationships.
	b.	ZIPs 6/10 identified to bind to members of mammalian prion protein family populate a common phylogenetic branch and represent, along with ZIP5, the subset of mouse ZIPs (out of fourteen ZIP paralogs) which objectively display the strongest sequence similarity to prion gene sequences.
	c.	A simple and plausible model for the emergence of the prion gene family in Chordata exists.

Source: Supporting Information of *PLoS ONE* 4(9): e7208. doi:10.1371/journal.pone.0007208
