# Supplementary material for: Evidence for Retrogene Origins of the Prion Gene Family
Source: PLoS One. 2011 Oct 27;6(10):e26800. doi: 10.1371/journal.pone.0026800 (PMC3203146; doi:10.1371/journal.pone.0026800)
Supplement: Table S2 — Protein accession numbers and abbreviations of species names. (PDF) [file pone.0026800.s005.pdf]

Table S2. Protein accession numbers and abbreviations of species names

SEQUENCES	Ensembl	Entrez
**Sp_ZIP**		NP_594942.1
**Sc_YKE4**		NP_012241.1
**Mb_ZIP**		XP_001750768.1
**Ta_ZIP**		XP_002108729.1*†
**Hm_ZIP12**		XP_002160698.1
**Aa_ZIP**		XP_001648141.1
**Ag_ZIP**		XP_317935.4
**Dm_ZIP (1)**		NP_001097608.1
**Dm_ZIP (2)**		NP_523974.3
**Bf_ZIP (1)**		XP_002594126.1
**Bf_ZIP (2)**		XP_002608436.1
**Ci_ZIP**	ENSCINP00000006884	
**Dr_PrP-rel3**	ENSDARP00000017287	
**Dr_ZIP5**		XP_690258.2
**Dr_ZIP6**		NP_001001591.1
**Dr_ZIP8**	ENSDARP00000056149	
**Dr_ZIP10**		NP_956965.1
**Dr_ZIP14**		XP_001340102.2
**Tn_PrP1**		Q4SJ93
**Tn_ZIP6**		CAF89895.1
**Tr_PrP1**		AAN38988.1
**Tr_PrP2**		NP_001072109.1
**Tr_PrP-like**	ENSTRUP00000016346	
**Tr_ZIP8**	ENSTRUP00000045288	
**Tr_ZIP10**	ENSTRUP00000007404	
**Tr_ZIP12**	ENSTRUP00000019247	
**Tr_ZIP14**	ENSTRUP00000038591	
**Ol_ZIP10**	ENSORLP00000011004	
**Ga_PrP1**		CAL64057.1
**Ga_PrP2A**	ENSGACP00000007465	CAL64056.1
**Xl_PrP**		NP_001082180.1
**Xt_ZIP5**		NP_001120272.1
**Ts_PrP**		Q9I9C0
**Gg_PrP**	ENSGALP00000040285	
**Gg_ZIP10**	ENSGALP00000012604	
**Md_PrP**	ENSMODP00000014477	
**Md_ZIP6**	ENSMODP00000000564	
**Mm_Dpl**		AAF02544.1
**Mm_PrP**	ENSMUSP00000088833	
**Mm_ZIP4**	ENSMUSP00000073134	

**Mm_ZIP5**	ENSMUSP00000037753
**Mm_ZIP6**	ENSMUSP00000064667
**Mm_ZIP8**	ENSMUSP00000029810
**Mm_ZIP10**	ENSMUSP00000027131
**Mm_ZIP12**	ENSMUSP00000080911
**Mm_ZIP14**	ENSMUSP00000066108
**Hs_PrP**	ENSP00000368752
**Hs_ZIP4**	ENSP00000276833
**Hs_ZIP5**	ENSP00000266980
**Hs_ZIP6**	ENSP00000269187
**Hs_ZIP8**	ENSP00000378310
**Hs_ZIP10**	ENSP00000352655
**Hs_ZIP12**	ENSP00000366586
**Hs_ZIP14**	ENSP00000370635

Aa → *Aedes aegypti* (yellow fever mosquito)
Ag → *Anopheles gambiae* (African malaria mosquito)
Bf → *Branchiostoma floridae* (Florida lancelet)
Ci → *Ciona intestinalis* (vase tunicate)
Dm → *Drosophila melanogaster* (fruitfly)
Dr → *Danio rerio* (zebrafish)
Ga → *Gasterosteus aculeatus* (three-spined stickleback)
Gg → *Gallus gallus* (chicken)
Hm → *Hydra magnipapillata* (hydra)
Hs → *Homo sapiens* (human)
Mb → *Monosiga brevicollis* (marine choanoflagellate)
Md → *Monodelphis domestica* (gray short-tailed opossum)
Mm → *Mus musculus* (house mouse)
Ol → *Oryzias latipes* (medaka)
Sc → *Saccharomyces cerevisiae* (baker's yeast)
Sp → *Schizosaccharomyces pombe* (fission yeast)
Ta → *Trichoplax adhaerens*
Tn → *Tetraodon nigroviridis* (spotted green pufferfish)
Tr → *Takifugu rubripes* (Japanese pufferfish)
Ts → *Trachemys scripta* (red-eared slider turtle)
Xl → *Xenopus laevis* (African clawed frog)
Xt → *Xenopus tropicalis* (Western clawed frog)

*Exon 1 identified using an alignment of TRIADDRAFT_62928 and TRIADDRAFT_18721 genes.

†An additional CFC-containing ZIP sequence was identified in *Trichoplax adhaerens* (TRIADDRAFT_28345, CFC = CYSASQIFNIFKVSSTGADVKQFNSLSPALVQQVATSAC) based on an alignment with TRIADDRAFT_18721. However, due to incomplete exon annotation, the sequence was not used in the present analyses.
